# Pain Relieving Effect of Thermoplastic Lumbosacral Orthosis with Adjustable Posterior Pad in Chronic Non-Specific Low Back Pain

**Published:** 2011-12-01

**Authors:** Y Salekzamani, S Mirzaee, S K Shakouri, N Nezami

**Affiliations:** 1 Physical Medicine and Rehabilitation Research Center, Tabriz, Eastern Azerbaijan, Iran; 2 Drug Applied Research Center, Tabriz University of Medical Sciences, Tabriz, Eastern Azerbaijan, Iran

**Keywords:** Orthosis, Lumbosacral, Thermoplastic, Low back pain

Dear Editor,

Low back pain (LBP) is one of the common disabling conditions experienced by individuals through the world and the lifetime prevalence of LBP was reported about 84%.[1][2] A type of LBP which occurs in the absence of an identifiable cause, is called non-specific LBP.[3] Non-specific LBP is managed conservatively by physical therapy and in many cases by applying orthosis. A wide variety of orthotic designs, ranging from lumbosacral corsets to rigid thermoplastic thoracolumbosacral orthosis are used for controlling LBP.[4]

During the present prospective study,25 male patients with chronic non-specific chronic LBP were evaluated after five days trial of a thermoplastic lumbosacral orthosis with a posterior adjustable pad. Inclusion criteria were LBP for 12 months or longer; having a previous history of routine LBP treatments including rest, physical therapy, lumbosacral corset without complete pain relief; non-specific findings in previous para-clinical evaluation. Ethical approval of our research was given by the Ethics Committee of the Tabriz University of Medical Sciences.

**Fig. 1:**
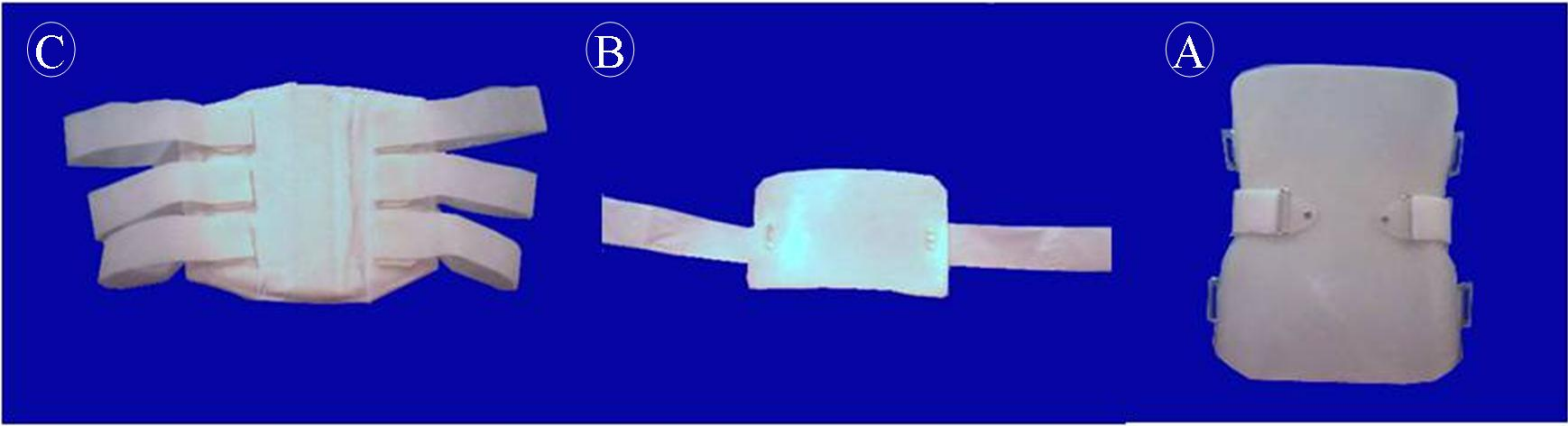
Different parts of lumbosacral orthosis with adjustable posterior pad. (A) posterior part, anterior part or abdominal pad, and posterior adjustable lumbar pad. Posterior part, fabricated out of thermoplastic materials, composed the body of orthosis. In the thoracic part, the superior edge of orthosis rests on 24 mm below the inferior angle of the scapulae bone. In the pelvic part, the inferior edge of orthosis rests on the sacrococcygeal junction in the midline. (B) Posterior adjustable lumbar pad of orthosis, the most important part, is connected to the posterior part with straps and located above the iliac crest. This adjustable pad may be moved and located in the desired lordotic positions regarding different tensions on the straps. (C) Anterior part of the orthosis, located between xiphoid process and pubic symphysis, is fabricated out of soft materials, on contrary to the posterior part, and fastened to the posterior part with velcro straps

The thermoplastic lumbosacral orthosis with a posterior adjustable pad, used in present study, had three parts (Figure 1). This adjustable pad could be moved and located in the desired lordotic positions regarding different tensions on the straps with the advantage include low weight and being comfortable. After adjusting the orthosis, patients were asked to have their usual activities such as walking and going up and down stairs for 30 minutes. Patients who did not have any problem during this 30 minutes, were asked to use the orthosis for the next five days during which they kept their usual daily activity and did not use any type of medication.

The severity of pain was measured using a modified visual analog scale (VAS) scoring method. A VAS is a 10 cm horizontal line ranged from no pain to severe pain. The patient marked the point that he felt representing his perception of current state. Patients’ LBP severity was assessed by using VAS at baseline (VAS-A) and 5 days (VAS-B) after using fitted lumbosacral orthosis. At the times of assessing VAS-A and VAS-B, a single standing lateral radiograph without orthosis was obtained from the lumbosacral region of patients to determine the lumbar lordosis angle (LLA) and lumbosacral angle (LSA). Statistical analyses were performed using the SPSS Statistical Package (Version 13.0, Chicago, IL, USA). The results are shown as mean±standard deviation (SD). Then, general linear model repeated measures test was used to assess the difference after using orthosis. A p value less than 0.05 was considered significant.

The mean age, body mass index and duration of LBP were 45.92±8.13 years, 24.1±0.78 kg/m(2), and 2.36±1.25 years, respectively. Orthosis significantly affected VAS scores, Wilks’ Lambda was 0.190, F (2, 23) was 49.157 (p<0.001) and multivariate partial eta squared was 0.810. The VAS-A and VAS-B were 7.12±0.97 and 3.04±1.85, respectively. LBP severity significantly reduced after 5 days use of orthosis (p=0.001).

The LLA before and five days after using orthosis was respectively 45.44±7.07 and 47.20±6.29 degrees which did not significantly change. The LSA before and five days after using orthosis was 37.68±4.26 and 38.96±3.78 degrees, respectively, showing insignificant changes. VAS scores five days after application of orthosis, in comparison to the baseline scores, showed a significant decrease. In the present study, a different type of orthosis was used (thermoplastic, light and comfortable in nature; Figure 1). This type of orthosis may be produced in different sizes and pre-production forms may be prescribed for every single patient.

Several mechanisms were suggested for the pain relieving action of lumbosacral orthosis including motion restriction/decreased weight load on the spinal column, reduced abnormal pressure, reduced muscle fatigue and increased proprioception.[5][6] People with flat back developed several compensatory mechanisms to maintain an efficient gait and decreased joint damage, but these safeguards failed over the time. Flat back of orthosis not only caused backache, abnormal posture and abnormal body mechanics, but also compromised the stability of gait and function of the knee and hip joints.[7] So, preservation of physiologic lumbar lordosis was an important consideration during performing fusion of the lumbar spine.[8] In case of the present orthosis, setting of the lumbar lordosis, characterized for each individual, in a special position may have a pain relief effect on patients with non-specific LBP. Insignificant change of LLA and LSA after using orthosis showed functional effect of orthosis on the spinal column, instead of anatomical changes.

The present study results were affected by some potential limitations, which were only male patients, lack of a control group and short period of follow-up. For confirming, further studies on the large number of patients for a longer period of observation may be needed. Preliminary results of this study showed that using thermoplastic lumbosacral orthosis with adjustable posterior pad may have a pain relieving effect on patients with non-specific LBP and may improve their quality of life.
